# The Role of Dermcidin Isoform 2: A Two-Faceted Atherosclerotic Risk Factor for Coronary Artery Disease and the Effect of Acetyl Salicylic Acid on It

**DOI:** 10.1155/2012/987932

**Published:** 2012-02-06

**Authors:** Rajeshwary Ghosh, Uttam K. Maji, Rabindra Bhattacharya, Asru K. Sinha

**Affiliations:** ^1^Sinha Institute of Medical Science & Technology, 288 Kendua Main Road, Garia, Kolkata 700 084, India; ^2^Calcutta Medical College and Hospital, Kolkata 7000 73, India

## Abstract

Hypertension and diabetes mellitus are considered to be two major atherosclerotic risk factors for coronary artery disease (CAD). A stress-induced protein identified to be dermcidin isoform 2 of Mr. 11 kDa from blood plasma of hypertensive persons when injected (0.1 *μ*M) in rabbits increased the systolic pressure by 77% and diastolic pressure by 45% over the controls within 2 h. Ingestion of acetyl salicylic acid (150 mg/70 kg) by these subjects reduced systolic (130 mm Hg) and diastolic pressures (80 mm Hg) with reduction of plasma dermcidin level to normal ranges (9 nM). The protein was found to be a potent activator of platelet cyclooxygenase and inhibited insulin synthesis. Aspirin was found to reduce hypertension by reduction of plasma dermcidin level, neutralized the effect of cyclooxygenase, and restored the pancreatic insulin synthesis through NO synthesis. These results indicated that dermcidin could be a novel atherosclerotic risk factor for its hypertensive and diabetogenic effects.

## 1. Introduction

Acute ischemic heart disease (AIHD), a life-threatening condition, has been reported to be developed as a result of formation of thrombus due to the aggregation of platelets on the site of atherosclerotic plaque rupture on the wall of coronary artery [1]. The aggregation of platelets by itself is a life-saving process in the blood coagulation through the formation of prothrombinase complex on the activated platelet surface [2]. In contrast, extensive platelet aggregation on the site of the atherosclerotic plaque rupture resulted in the precipitation of AIHD [1]. And, as such, the atherosclerosis plays a critically important role in the genesis of prothrombotic condition leading to AIHD [3]. Indeed, in the absence of atherosclerosis, the platelet aggregation in many cases could be considered as a beneficial physiologic event. Diabetes mellitus (both type I and type II) and hypertension are considered to be the two major risk factors for prothrombotic condition leading to AIHD. Dyslipidemia, hyperhomocysteinemia, dysregulation of blood coagulation and fibrinolysis and inflammatory reaction are also reported to be atherosclerotic risk factors albeit lesser importance [4].

Although stress has been reported to instigate both diabetes mellitus [5] and hypertension [6], the interaction between these two risk factors remains obscure and speculative [7] except that hypertension is found to be associated with insulin resistance and dyslipidemia [8].

While the mechanism of insulin-induced hypertension remains obscure, insulin resistance itself is known to cause diabetic dyslipidemia. On the other hand, insulin itself has been reported to be a potent fibrinolytic agent both *in vivo* and *in vitro *through the formation of NO [9], and diabetes mellitus itself as a consequence may impair fibrinolysis [10]. In other words, many of the prothrombotic risk factors can arise due to impairment of the insulin effects [11].

A stress-induced oxidative protein, identified to be dermcidin isoform 2 (dermcidin) that has been reported to appear in the circulation in AIHD, is also found to be a potent platelet aggregating agent [12]. In a follow-up study, it was found that dermcidin was a potent inhibitor of insulin-induced NO synthesis in endothelial cells. As NO is reported to be a global vasodilatory agent [13] and the endothelial NO synthesis would play a critically important role in the control of hypertension [13], experiments were carried out to determine the role of dermcidin on the development of hypertension in animal model and in subjects suffering from systemic hypertension.

We report herein that the oxidative stress protein which was found to be a powerful inducer of platelet aggregation [12] not only inhibited insulin synthesis in the pancreatic *β* cells but also was a potent inhibitor of the hormone synthesis in the hepatocytes in the liver which has been reported to be an important source of the extrapancreatic hormone synthesis [14].

We also report that aspirin was found to inhibit the proatherosclerotic activities of dermcidin by restoring the synthesis of insulin as well as by normalizing the elevated blood pressure through the reduction of plasma level of the oxidative protein level induced by the systemic stimulation of NO synthesis.

## 2. Methods

### 2.1. Ethical Clearance

The protocol was approved by the Internal Review Board, Sinha Institute of Medical Science and Technology, Kolkata. All participants were asked to sign informed consent form. This study also used adult New Zealand rabbits. Appropriate permission was also obtained from the IRB.

### 2.2. Chemicals

Goat anti-rabbit immunoglobulin G-alkaline phosphatase was obtained from Sigma Chemical Co. Aspirin was obtained from Medica Zydus Healthcare. Maxisorp plates were from Nunc, Roskilde, Denmark. All other chemicals were of analytical grade.

### 2.3. Selection of Hypertensive Persons

All participating volunteers in the study came to Kolkata Medical College and Hospital, Kolkata as “outdoor” patients. Equal number of male and female volunteers with hypertension (*n* = 74, in each group) participated in the study. These hypertensive patients came to the hospital with minor ailments often with clinically undefined malaise. They were between the ages of 25 and 65 years. At presentation, none of the patients were aware of the fact that they had elevated blood pressures (BPs), and, as such, they never received any treatment for the condition. Any of these subjects who had systolic BP ≥ 140 mm of Hg and diastolic BP ≥ 90 mm of Hg was considered to be hypertensive [15] and was included in the study without any regard to the underlying etiologic mechanism involved in the development of the condition. The BPs were measured by sphygmomanometer.

#### 2.3.1. Exclusion Criteria for Hypertensive Patients

Patients with history of diabetes mellitus or with any life-threatening infection or cardiovascular/cerebrovascular conditions were excluded from the study. Care was also taken to exclude patients who were hospitalized for any condition within the last two months as well as the persons who were taking any medication including antihypertensive drug or aspirin.

### 2.4. Selection of Acute Myocardial Infarction (AMI) Patients

All patients (*n* = 29) between ages of 49 and 61 (median age 54 years) were admitted to the Intensive Care Unit of the Calcutta Medical College and Hospital, Kolkata.

These patients met the following criteria of AMI: they had chest pain characteristic of myocardial ischemia for 30 mins or more and the electrocardiogram (ECG) showed ST segment elevation of at least two leads in the ECG reflecting a single myocardial region. The confirmation of the condition was determined by the elevated creatine kinase and creatine kinase-MB isoenzyme assay in the blood plasma. The sampling of blood was made within 6 h of the onset of the anginal attack before any therapy for the condition was initiated. Only those AMI patients who refused to ingest aspirin due to personal/religious beliefs served as “controls” when necessary.

#### 2.4.1. Exclusion Criteria

(1) Patients with the history of diabetes mellitus, (2) showing the presence of bundle branch block or left ventricular hypertrophy in the ECG (3) or suffering from any severe infection, (4) took aspirin at least within 2 weeks, (5) hospitalized for any condition within two months, and (6) took any cardiac medication including any antihypertensive drug within last 21 days were excluded from the study.

### 2.5. Selection of Normal Subjects

Age- and sex-matched normal volunteers (*n* = 74) also participated in the study. Selected volunteers had normal kidney functions as determined by their plasma creatinine (<1 mg/dL) and urea (6–17 mg/dL) levels. The urinary excretion of protein in the normal participants was <125 mg/day. The HDL and LDL levels were also within normal limits. No female volunteers had ever taken any contraceptive medication.

### 2.6. Collection of Blood

Blood samples (20–25 mL), obtained from the participants by venipuncture by using 19-gauge siliconized needles, were collected in plastic vials and anticoagulated by gently mixing 9 vol of the blood with 1 vol of 0.13 mM sodium citrate [16]. The cell-free plasma (CFP) was prepared by centrifuging the blood sample from the participants at 30,000 g for 30 min at 0°C.

### 2.7. Identification of Dermcidin in the Cell-Free Plasma Sample from Hypertensive Patients

As mentioned before, a new plasma protein of Mr 11 kDa was found to be present in the CFP of the AMI patients [12]. As described under Section 3, to determine whether this protein might also be present in the plasma of hypertensive persons, when the CFP from hypertensive plasma was subjected to SDS- polyacrylamide gel electrophoresis [17], a novel protein band of Mr. 11 kDa was found to be present in the gel in the case of hypertensive CFP compared to that in the CFP from normal volunteers. This protein band of Mr 11 kDa was next excised from the gel, triturated in 0.9% NaCl, and clarified by centrifugation. The clarified sample was reelectrophoresed on polyacrylamide gel in the absence of SDS. The staining of the gel demonstrated the presence of a single band. The staining of an identical gel with AgNO_3_ [18] failed to show the presence of any other band in the gel. The 11 kDa band in an identical gel, not stained with AgNO_3_ was excised out of the gel, triturated in 0.9% NaCl, and dialyzed overnight at 4°C against 0.9% NaCl. The final gel slices were washed twice with 50% high-performance liquid chromatography grade acetonitrile in water for 2-3 min with gentle shaking and discarding the supernatant after each wash. The amino acid sequence of the protein sample thus prepared was determined by Mass Spectrometry and Proteomic Resource Core, Harvard University using microcapillary reverse-phase HPLC nanoelectrospray tandem mass spectrometry (*μ*LC/MS/MS) on a Thermo LTQ-Orbitrap mass spectrometer. The protein was identified to be dermcidin isoform 2, an oxidative stress protein, composed of 105 amino acids, that was previously found to be present in the plasma of AMI patients [12].

### 2.8. Preparation of Dermcidin

Dermcidin used for its biological activity was prepared by sequential polyacrylamide gel electrophoresis in the presence and absence of SDS as described above. The isolated protein preparations was pooled and concentrated by using polyethylene glycol as described [19]. Before use, the concentrated preparation was dialyzed overnight at 4°C against 0.9% NaCl.

### 2.9. Measurement of BP in Animal Model

Normal adult New Zealand white rabbits of either gender were used for the study. To ensure that the animals were disease free, they were subjected to a thorough checkup by a licensed veterinarian. A mercury sphygmomanometer was used to record the BPs of the animals [20].

### 2.10. Assay of NO Synthesis

The synthesis of NO was determined by methemoglobin method [21] as described before. The validity of the assay was confirmed by an independent chemiluminescence method [22].

### 2.11. Enzyme-Linked Immunosorbent Assay (ELISA) of Dermcidin

Polyclonal antibody against pure dermcidin was raised in adult New Zealand rabbit by intradermal injection of dermcidin emulsified with Freund's adjuvant as described [12].

The feasibility of the determination of dermcidin by ELISA [23] was tested by immunoblot technique [24]. The analytical precision of the assay for dermcidin by ELISA as determined by “recovery” experiments was found to be >90%.

### 2.12. ELISA of Insulin

Insulin synthesized in the pancreatic islets of Langerhans was quantitated by ELISA as described before [12, 23].

### 2.13. Oral Administration of Aspirin

In some phase of this study, the selected subjects were asked to take an adequate meal consisting of carbohydrate rich food like bread as well as foods rich in protein like meat (90 gm/70 kg body weight), milk, and cheese and then swallow a 150 mg aspirin tablet with water.

### 2.14. The Preparation of Platelet-Rich Plasma and the Determination of Platelet Aggregation

The platelet-rich plasma (PRP) was prepared from the anticoagulated blood by centrifuging the sample at 200 g as described [16]. The aggregation of platelets of normal volunteers was carried out in a platelet aggregometer (SEAC Clot 2S) using ADP as the aggregating agonist as described before [16]. The aggregation of platelets in PRP of these normal volunteers was also studied using different concentrations of dermcidin as an aggregating agent in a similar way as in the case of ADP.

### 2.15. Determination of Thromboxane A_**2**_ Synthesis in Platelets

The production of thromboxane A_2 _ was determined by radioimmunoassay of thromboxane B_2_ [16]. After the aggregation of platelet was completed (5 min) at 37°C, the formation of thromboxane B_2_ was assayed to determine the synthesis of thromboxane A_2_.

### 2.16. The Preparation of Goat Carotid Artery Endothelial Cell Homogenate

Endothelial cells were prepared from the carotid artery of the freshly slaughtered goat as described before [25]. The endothelial cell suspension in Tyrod's buffer pH 7.4 was disrupted by repeated freezing and thawing the cell suspension (20 mg/mL) in liquid N_2 _ [25]. The disrupted cell mass was centrifuged at 60,000 g for 30 min at 0°C. The supernatant was used as the source of endothelial nitric oxide synthase (eNOS).

### 2.17. Lineweaver-Burk Plot of Insulin-Activated eNOS in the Endothelial Cell Homogenate in the Presence or Absence of Dermcidin

The reaction mixture contained different concentrations of *l*-arginine in the presence of 2 mM CaCl_2_ with 100 *μ*units of insulin/mL and in the presence or absence of 0.1 *μ*M dermcidin in Tyrod's buffer pH 7.4 in a total volume of 1.0 mL. After 20 min of incubation at 37°C under N_2_, (during the steady state of formation of NO (1–30 min)) the product NO was determined as described before [21].

### 2.18. Preparation of the Islets of Langerhans from the Mice Pancreas and the Determination of the Effect of Dermcidin on the Glucose-Induced Synthesis of Insulin in the Pancreatic Islets of Langerhans

In some phases of the study, it was necessary to determine the glucose-induced synthesis of insulin using the islets of Langerhans. The islets of Langerhans were prepared and suspended in Krebs' bicarbonate buffer (pH 7.4) as described [12] and were incubated with or without 0.02 M glucose in the presence and absence of 0.1 *μ*M dermcidin at 37°C for 0–30 min, and the synthesis of insulin was determined by *in vitro* translation of the mRNA [26]. Insulin produced was determined by ELISA as described above.

### 2.19. Statistical Analysis

The significance of the results obtained was analyzed by Student's *t*-test. Significance *P* < 0.0001 is considered to be significant. Correlation coefficient, Pearson score “*r*”, is such that −1 ≤ *r* ≤ +1 is considered to be acceptable. The (+) and (–) signs are used for positive linear correlations and negative linear correlations, respectively. Where appropriate, the significance of the results was verified by nonparametric Mann Whitney *U* test.

## 3. Results

### 3.1. Appearance of Dermcidin in the Circulation of Hypertensive Patients

**Figure 1:**
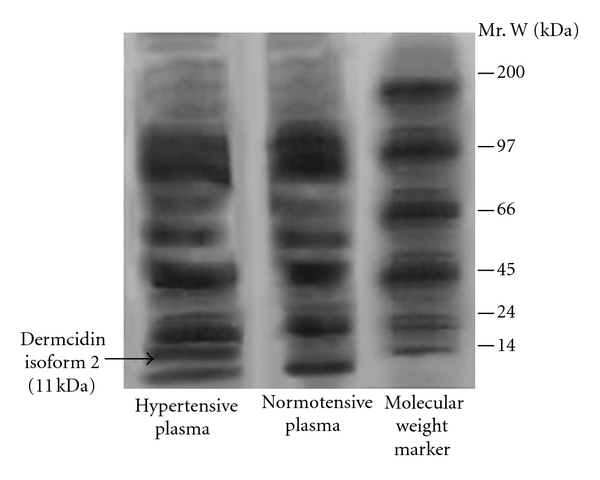
Sodium dodecyl sulfate polyacrylamide gel electrophoresis of the cell-free plasma from hypertensive and normotensive subjects. Cell-free plasma (CFP) was prepared from the blood samples of hypertensive and normotensive subjects and electrophorosed in SDS-polyacrylamide. The protein band was stained by Coomassie brilliant blue as described in Section 2. The arrow indicates the position of 11 kDa protein band. The figure represents the typical gel electrophoresis of the CFP from at least 10 different hypertensive and normotensive subjects.

As mentioned earlier, there might be likelihood that the stress-induced protein(s) might play a role in the genesis of systemic hypertension. To verify this possibility of the presence of any novel protein(s) in the circulation of hypertensive patients which may result in the elevation of the BPs, the plasma sample of the hypertensive patients was electrophorosed on SDS-polyacrylamide gel. The staining of the gel with Coomassie Brilliant Blue revealed the presence of a new protein band of Mr 11 kDa in the plasma of hypertensive patients compared to that in normal controls (Figure 1). As described in Section 2, repeated gel electrophoresis of the excised protein band from the hypertensive subjects in the absence of SDS was analyzed to determine the amino acid sequence which demonstrated that the novel protein was comprised of 105 amino acids. The protein database matching identified the protein to be dermcidin isoform 2 (hereafter referred to as dermcidin only), which has been reported before to be an oxidative stress protein [12].

### 3.2. Effect of Dermcidin on the Blood Pressure and NO Levels in the Animal Model

**Figure 2:**
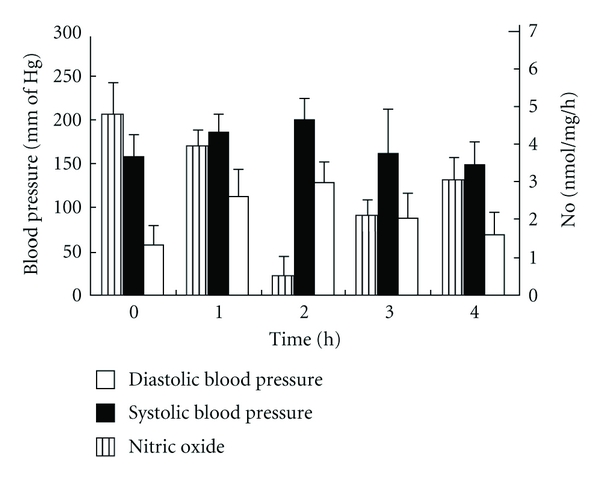
The effect of injection of dermcidin to the rabbits on the systolic and diastolic pressures and on the plasma NO level at different times after the injection of the protein. The electrophoretically purified dermcidin was injected to the circulation of “normal” rabbits (1.0 nmol/kg body weight). Both systolic and diastolic pressures and the plasma NO levels were determined at different time after the administration of the oxidative stress protein. The results shown are mean ± SD of six different experiments 3 times each using 6 different rabbits.

Experiments were carried out to determine the effect of dermcidin on the systemic blood pressure levels, if any, in animal model using rabbit. The oxidative stress protein was injected (1.0 nmol/kg body weight) in the ear vein of the test animals, and the blood pressures were recorded at different time intervals. It was found that the venous injection of dermcidin (1.0 nmol/kg body weight) in the animal resulted in the elevation of basal systolic pressure of 155 ± 4.78 mm of Hg to 200 ± 10 mm of Hg with simultaneous increase of diastolic pressure of 57.5 ± 8.66 mm Hg to 125 ± 5.77 mm Hg after 2 h of the injection (*P* < 0.0001, *n* = 5) (Figure 2). However, it was also noted that after 2 h there was a gradual reduction in the level of the elevated BP in this animal model reaching to *≈*“normal” ranges (i.e., the BPs at the pretreatment level) of 147 ± 5.77 mm Hg (systolic) and 69.15 ± 10 mm Hg (diastolic) at 4 h. These results suggested that the increase of dermcidin (1.0 nmol/kg body weight) in the animal model led to an acute elevation of BPs which persisted for 2 h, and thereafter there was a gradual normalization of the elevated BPs in the test animal.

It has been reported before that increase in BP levels was associated with the reduction of systemic nitric oxide (NO) level [27]. Studies were carried out to determine the effect of dermcidin on the plasma NO level in the test animals. When dermcidin (*≈*1 *μ*M) was injected in the test animals, and blood samples were collected from the ear vein to determine NO level in the plasma at different time intervals, it was found that the basal NO level prior to dermcidin injection, which was 4.8 ± 0.127 nmol/mL, was reduced to 0.447 ± 0.017 nmol/mL (*P* < 0.0001, *n* = 5) at 2 h (Figure 2). However, after 2 h, there was a gradual retrieval of the systemic NO level which reached to *≈*“normal” ranges (3.5 nmol) at 4 h. The plasma dermcidin level in the rabbits that was found to increase to 98.48 ± 10.8 nM after the injection of the protein at 2 h was simultaneously found to decrease to 33.78 ± 7.44 nM at 4 h (*P* < 0.0001).

In the control experiment, equal numbers of animals were administered with equal volume of 0.9% NaCl solution. In contrast to dermcidin, the vehicles alone had no effect either on the pressure or NO.

### 3.3. Plasma Dermcidin Level in Hypertensive and Normotensive Subjects

As the above results indicated that dermcidin might be involved in the increase of systemic blood pressures, the plasma levels of the oxidative stress protein in the circulation of hypertensive subjects (*n* = 74) as well as in equal number of normotensive subjects were compared to determine the correlation between the BPs level and plasma dermcidin level. It was found that the plasma dermcidin level in hypertensive subjects was 98 pmol/mL (median ranging between 43.1 to 175 pmol/mL) which was significantly higher compared to normotensive subjects [5 pmol/mL (median ranging between 0–24 pmol/mL)]. Mann Whitney *U* test between the dermcidin levels in these two groups showed that the *P* (significance) value was <0.0001. The coefficient of correlation “*r*” between the plasma dermcidin and BP levels was determined to be highly and positively correlated (Table 1).

### 3.4. The Effect of Reduction of Plasma Dermcidin Level on the Systemic Blood Pressure in Hypertensive Subjects

**Figure 3:**
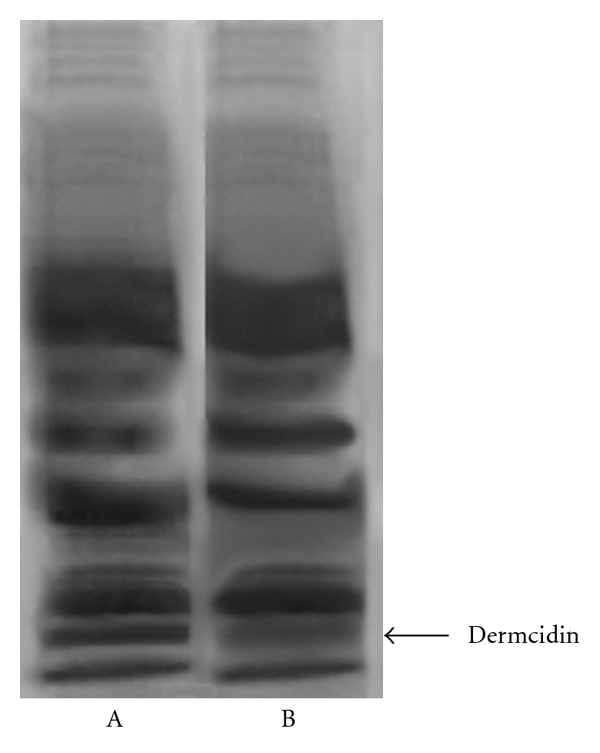
SDS gel electrophoresis of plasma of hypertensive patients before and after the ingestion of aspirin. The plasma from the hypertensive patients was subjected to SDS polyacrylamide gel electrophoresis before (Lane A) and after (Lane B) the ingestion of aspirin. As described in Section 2, patients were asked to swallow 150 mg aspirin. After 3 h of the ingestion of the compound, CFP was prepared and SDS gel electrophoresis was run and subsequently stained with Coomassie Brilliant Blue. Please note that the intensity of the 11 kDa protein band was less dense (Lane B) compared to before the ingestion of aspirin (Lane A). The figure shown here is a typical representative of at least 10 more identical experiments.

It has been reported before that the oral ingestion of acetyl salicylic acid (aspirin) resulted in the reduction of dermcidin level through the increase of systemic NO level [12]. As the results described above indicated that the increase of plasma dermcidin level could lead to the increase of systemic BPs that was associated with the reduction of plasma NO level, studies were conducted to determine whether the reduction of dermcidin level could actually result in the reduction of systemic BPs in hypertensive subjects. As the oral ingestion of aspirin has been reported to decrease the plasma dermcidin level [12], the participating hypertensive subjects (*n* = 74) were asked to ingest 150 mg aspirin as described in Section 2. It was found that, 3 h after the oral ingestion of aspirin, the plasma dermcidin level in these hypertensive subjects decreased from 98 nM (median, ranging from 43.1 nM to 175 nM) to 19.1 nM (median, ranging from 2.9 nM to 51 nM) with decrease of both systolic (160 to 130 mm of Hg (Median)) and diastolic pressures (90 to 80 mm of Hg (Median)) (Table 2). SDS polyacrylamide gel electrophoresis of the plasma from the hypertensive patients who had ingested aspirin showed a markedly less intense band of dermcidin (Mr 11 kDa) when compared to patients who did not undergo aspirin treatment (Figure 3).

### 3.5. ADP and Dermcidin-Induced Platelet Aggregation and the Stimulation of Thromboxane A_2_ Synthesis

**Figure 4:**
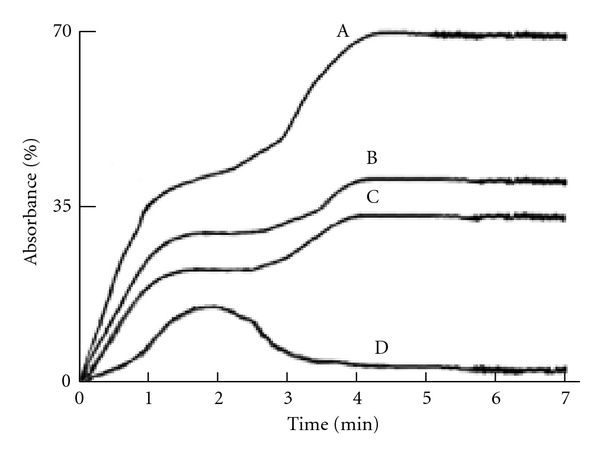
Aggregation of platelets by ADP and dermcidin and the aggregation of platelets in the presence of both ADP and dermcidin added to PRP. Platelet-rich plasma was prepared from the blood of normal volunteers, and the aggregation of platelet was determined by treating the PRP with either ADP (4 *μ*M) or dermcidin (0.1 *μ*M) or with both ADP and dermcidin. The curve A: aggregation of platelets when both ADP and dermcidin were added to the PRP. Curve B: dermcidin-induced platelet aggregation. Curve C: ADP-induced platelet aggregation. Curve D: aspirin-(80 *μ*M) induced inhibition of platelet aggregation induced by dermcidin. The figure represents a typical platelet aggregation in the presence of ADP, dermcidin, or both ADP and dermcidin from 6 different experiments using blood samples from 6 different normal volunteers.

Since hypertension has been reported to be a major risk factor for coronary artery disease, it was thought that the occurrence of this oxidative stress protein in hypertensive plasma could ultimately lead to coronary artery disease through increased platelet aggregation leading to thrombosis [12]. Experiments were carried out to determine whether the electrophoretically purified oxidative stress protein from the hypertensive plasma by SDS polyacrylamide gel electrophoresis could have any effect on the aggregation of platelets. The aggregation of platelets was studied by adding different concentrations of the purified dermcidin to PRP as described in Section 2. The dermcidin-induced platelet aggregation was compared to that induced by ADP, which is reported to be the most important platelet aggregating agent in the genesis of the coronary artery disease in man due to the aggregation of platelets in the coronary artery [2]. It was found that dermcidin (0.1 *μ*M), on mol/mol basis, was *≈*40-fold stronger platelet-aggregating agent compared to ADP (4.0 *μ*M) (*P* < 0.0001; *n* = 10) (Figure 4). Furthermore, like ADP, the oxidative stress protein was found to be a potent stimulator of thromboxane A_2_ synthesis in platelets through the activation of cyclooxygenase [16]. It was found that the treatment of normal PRP with 4 *μ*M ADP resulted in the synthesis of 21.3 ± 3.6 pmol of thromboxane A_2_/10^8^ platelets after 5 min of incubation at 37°C. The treatment of the same PRP with 0.1 *μ*M dermcidin resulted in the production of 29.2 ± 3.6 pmol thromboxane A_2_/10^8^ platelets under otherwise identical conditions (*P* < 0.0001; *n* = 10). Addition of dermcidin together with ADP showed “superaggregation of platelets” in the presence of 0.1 *μ*M dermcidin and 4.0 *μ*M ADP (Figure 4) that resulted in the stimulated production of 31.5 ± 5.22 pmol thromboxane A_2_/10^8^ platelets. These results indicated that dermcidin had an additive effect on the ADP-induced platelet aggregation with concomitant increase of thromboxane A_2_ synthesis. On the other hand, incubation of the platelet-rich plasma with 80 *μ*M aspirin, a well-known inhibitor of platelet cyclooxygenase [28], and ADP-induced platelet aggregation [29] was also capable of inhibiting the aggregation induced by dermcidin (Figure 4).

### 3.6. The Effect of Dermcidin on the Inhibition of Endothelial Nitric Oxide Synthase (eNOS)

As described in the results, the injection of dermcidin that led to the increase of BPs in the animal model was associated with the decrease of the plasma NO level (Figure 2). Nitric oxide, demonstrated to be the endothelial-derived vasorelaxing factor, has been reported to be a global antihypertensive agent [13]. And, as such, the dermcidin-induced reduction of plasma NO level could be suggested to result in the increase of systemic hypertension. However, eNOS by itself has been reported to have little or no basal enzymic activity for the production of NO, and this enzyme in the endothelial cells has been reported to be stimulated only in the presence of appropriate activators for the synthesis of NO leading to the control of the elevated BPs [13].

As only limited numbers of physiologic activators of eNOS are known [13] and since insulin has been reported to stimulate NO synthesis in various cells including endothelial cells [11], the dermcidin-induced inhibition of the insulin activated nitric oxide synthase leading to the inhibition of NO production from *l*-arginine was determined by adding dermcidin to the goat endothelial cell homogenate preparation treated with insulin in the presence of *l*-arginine (the substrate).

**Figure 5:**
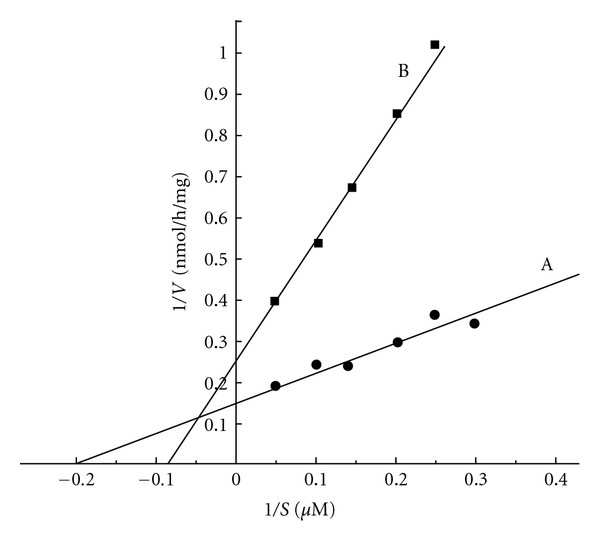
Lineweaver-Burk plot of the inhibition of nitric oxide synthase activated by insulin in the cell-free homogenate from the goat artery endothelial cells. The cell-free homogenate of the endothelial cells from the carotid artery of the goat was prepared as described in Section 2. Lineweaver-Burk plot was constructed by adding different amounts of *l*-arginine to the reaction mixture containing 100 *μ*units/mL insulin in the presence or absence of 0.1 *μ*M dermcidin. The line A represents the formation of NO in the presence of insulin, and the line B represents the formation of NO in the presence of both the insulin and dermcidin. Each point represents mean of 5 different experiments each in triplicate.

Lineweaver-Burk plot of the eNOS in the supernatant from the endothelial cell homogenate in the presence of insulin that resulted in the stimulation of NO synthesis in the reaction mixture as described in Section 2 demonstrated that the Km of eNOS was 9.43 *μ*M arginine with the maximum velocity (Vmax) of 7 nmol NO/h/mg. The addition of 0.1 *μ*M dermcidin to the reaction mixture increased the Km from 9.43 *μ*M to 26.3 *μ*M arginine with concomitant decrease of the Vmax from 7 nmol NO/h/mg to 3.8 nmol NO/h/mg indicating that the rate synthesis of NO in the presence of insulin was decreased by nearly 50% in the presence of 0.1 *μ*M dermcidin *in vitro* (Figure 5).

### 3.7. The Effect of Dermcidin on the Inhibition of Glucose-Stimulated Release and Synthesis of Pancreatic Insulin

As described above, dermcidin was a potent inhibitor of insulin activated nitric oxide synthase and thereby might play a critical role in the development of hypertension. Studies were further conducted to determine its role in the pancreatic synthesis of insulin. It was found that the addition of 0.02 M glucose to the incubation mixture containing the pancreatic islets of Langerhans resulted in the increase of insulin synthesis from the basal 0.012 ± 0.004 *μ*units insulin/mg/h to 0.088 ± 0.005 *μ*units insulin/mg/h. In contrast, the addition of 0.1 *μ*M dermcidin in the incubation mixture resulted in the inhibition of the insulin synthesis by 50% (0.006 *μ*units insulin/mg/h) (*P* < 0.0001; *n* = 5).

### 3.8. Effect of Administration of Aspirin on the Plasma Dermcidin and Insulin Level in Patients with Acute Myocardial Infarction (AMI)

It has been described above that the use of aspirin *in vivo *was capable of reducing the plasma dermcidin level due to the systemic increase of NO level (Table 2). To determine whether the ingestion of aspirin could have any effect on the plasma insulin level in AMI who had plasma insulin level of 15 *μ*units/dL (Median), all participating subjects with the condition (*n* = 29) were asked to ingest 350 mg of aspirin. It was found that the oral administration of aspirin in AMI patients resulted in the increase of plasma insulin level from 15 *μ*units/dL (median; ranging from 25 to 0 *μ*units/dL) before the ingestion of aspirin to 150 *μ*units/dL (median; ranging from 125 to 40 *μ*units/dL) after the ingestion of the compound within 24 h. It was also found that there was a concomitant decrease in the dermcidin level from 116 nM (median) (ranging from 72 nM to 173 nM) to 11 nM (median) ranging from 0 nM to 45 nM. The coefficient of correlation (*r*) between the plasma dermcidin and insulin level was determined to be −0.70 (without aspirin) and −0.721 (with aspirin) indicating that the plasma dermcidin and insulin levels were negatively correlated.

## 4. Discussion

As described above, an oxidative stress protein, determined to be dermcidin, was not only found to appear in the circulation of the hypertensive subjects (Figure 1), but the stress-induced protein of Mr 11 kDa was also found to be a potent inducer of platelet aggregation (Figure 4). Although it was not possible for us to demonstrate directly the effects of dermcidin in the increase of blood pressures in human subjects, the plasma dermcidin level in hypertensive subjects was highly correlated with both the systolic (*r* = +0.924) and diastolic (*r* = +0.909) blood pressure levels in hypertensive subjects (Table 1). Furthermore, the injection of the dermcidin itself in rabbits was found to acutely increase the blood pressure levels. The increase of blood pressure was found to be related to the decrease of plasma NO level induced by the injection of dermcidin in the circulation of the test animal. On the other hand, the decrease of the plasma dermcidin level in hypertensive volunteers by the oral administration of aspirin which has been reported to increase plasma NO level in humans [12, 16] resulted simultaneously in the reduction of both the systolic and diastolic pressures with concomitant decrease of plasma dermcidin level in the hypertensive subjects (Table 2). The correlation coefficient “*r*” (Pearson value) between the reduction of plasma dermcidin levels and the systolic and diastolic pressures was +0.689 and +0.846 respectively indicating that the reduction of these pressures was positively correlated to the reduction of the plasma dermcidin level. Thus, not only the increase of plasma dermcidin was highly correlated to the increase of both diastolic and systolic pressures in the hypertensive subjects but also the decrease of the plasma dermcidin level was highly correlated to the decrease of the blood pressures in these subjects. In separate experiments, it was found that the dermcidin-induced decrease of the plasma NO level was a consequence of the inhibition of a constitutive form of nitric oxide synthase (cNOS) activated by insulin in the endothelial cells from the carotid artery from goat (Figure 5). Nitric oxide, a global vasodilating agent [13], originally identified as “endothelial derived relaxing factor” [13], is known to be produced in the endothelial cells. However, contrary to the expectation, the endothelial nitric oxide synthase (eNOS) had no basal enzymic activity for the synthesis of NO and appropriate stimulator was needed for the synthesis of NO by the enzyme [13]. The numbers of physiologic stimulators for the eNOS currently reported are however limited. Only a kidney-cortex-derived hypotensive protein called renal cortexin [25] and insulin [11] are currently known to activate eNOS in the endothelial cells homogenate *in vitro*. As reported in Section 3, dermcidin was a potent inhibitor of insulin-induced activated eNOS that led to the inhibition of systemic NO synthesis in the endothelial cells homogenate. Lineweaver Burk plot of the dermcidin-induced effect on the insulin-activated eNOS demonstrated that dermcidin was a competitive inhibitor of the enzyme and was found to compete with *l*-arginine, the substrate of eNOS (Figure 5) for the synthesis of NO. Similar results were also obtained using renal cortexin (0.1 *μ*M) in the inhibition by dermcidin on the cortexin-activated eNOS of the endothelial cell homogenate (unpublished). These results suggested that dermcidin could act as systemic competitive inhibitor of NOS of the endothelial cells.

As described above, the aggregation of human blood platelets induced by dermcidin was induced by the activation of the platelet cyclooxygenase.

Although the effect of dermcidin in the increase of blood pressures lasted only for 4 h in the animal model, as the stress-induced protein, on mol/mol basis, was found to be 40 times more effective activator of cyclooxygenase when compared to that by ADP itself, dermcidin could be critically important in the development of CAD through thrombus in hypertension [12] even if the increase of plasma dermcidin level was only for a short time. It should be mentioned that the injection of dermcidin was capable of developing coronary artery disease in the animal model in the suboptimal amount of ADP within 30 min [12].

The development of atherosclerosis has been established to be the major pathological event leading to CAD [3]. Hypertension and diabetes mellitus are reported to be the two most important risk factors for atherosclerosis among other risk factors including dyslipidemia, hyperhomocysteinemia, dysregulation of blood coagulation, and fibrinolysis [4]. However, the biochemical mechanism involved in the hypertension-induced atherosclerosis remains obscure. As described in the results, not only dermcidin increased blood pressure and induced platelet aggregation leading to CAD through thrombogenesis, but also it has been found that the stress-induced protein was a potent inhibitor of insulin synthesis in the pancreatic *β* cells [12] as well as in the hepatocytes. In this context, dermcidin-induced inhibition of insulin was unique in that no other protein is currently known which is capable of inhibiting pancreatic insulin synthesis. The hypertensive protein could potentially be a double-edged risk factor for atherosclerosis leading to CAD through the development of both hypertension and diabetes mellitus. The inhibition of insulin-induced NO synthesis in the endothelial cells (Figure 5) could result itself in hypertension. Although the systemic decrease of NO level would be expected to result in the increase of BPs [30], no physiologic inhibitor of NO synthesis in humans has yet been reported that may initiate the increase of systemic BPs. In this sense, dermcidin, a protein produced due to stress, was unique in its role in the development of hypertension.

## 5. Conclusion

Close association between insulin resistance and hypertension has been reported before [30]. Our studies revealed a relation between hypertension and diabetes mellitus leading to the development of atherosclerosis. Dermcidin, which led to the inhibition of pancreatic insulin synthesis as well as the inhibition of insulin-induced NO synthesis, seems to offer a possible link between the two major risk factors leading to atherosclerosis due to repeated exposure to stress, and the stress-induced protein might actually precipitate CAD through the activation of platelet cyclooxygenase leading to platelet aggregation.

## Figures and Tables

**Table 1:** Correlation between plasma dermcidin level and systolic and diastolic pressures in normotensive and hypertensive subjects.

Normotensive subjects					Hypertensive subjects			
Parameters	Dermcidin(pmol/mL)	Systolic blood pressure (mm of Hg)	Diastolic blood pressure (mm of Hg)	NO (nmol/h)	Dermcidin(pmol/mL)	Systolic blood pressure (mm of Hg)	Diastolic blood pressure (mm of Hg)	NO (nmol/h)
Range	0–24	115–130	75–85	4.0 ± 1.4	43.1–175	150–180	85–110	0.4 ± 0.19
Median	5	125	80		98	160	90	

“Pearson *r*” (correlation coefficient) = + 0.922 and + 0.844 between dermcidin level and systolic and diastolic pressures, respectively, in normotensive subjects.

“Pearson *r*" (correlation coefficient) = + 0.924 and + 0.909 between dermcidin level and systolic and diastolic pressures, respectively, in hypertensive subjects.

The significance (*P* value) was *P* < 0.0001 between dermcidin levels, systolic and diastolic pressures in the normotensive and hypertensive subjects as determined by the Mann Whitney *U* test with the medians significantly different.

Blood samples were collected from both normotensive and hypertensive subjects (*n* = 74 in each group) by venipuncture as described in Section 2. The plasma dermcidin level was determined by ELISA by using electrophoretically purified dermcidin as described. The blood pressures were determined by sphygmomanometer at presentation.

**Table 2:** Effect of oral ingestion of aspirin on the blood pressures and on the dermcidin levels in hypertensive subjects.

Hypertensive subjects								
Before aspirin ingestion					After aspirin ingestion			
Parameters	Dermcidin(pmol/mL)	Systolic blood pressure (mm of Hg)	Diastolic blood pressure (mm of Hg)	NO (nmol/h)	Dermcidin (pmol/mL)	Systolic blood pressure (mm of Hg)	Diastolic blood pressure (mm of Hg)	NO (nmol/h)
Range	43.1–175	150–180	85–110	0.4 ± 0.19	2.9–51	115–140	75–85	1.9 ± 0.5
Median	98	160	90		19.1	130	80	

“Pearson *r*" (correlation coefficient) = + 0.924 and *= + *0.909 between dermcidin level and systolic and diastolic pressures, respectively, before aspirin ingestion.

“Pearson *r*" (correlation coefficient) = + 0.689 and + 0.846 between dermcidin level and systolic and diastolic pressures, respectively, after the ingestion of aspirin.

The significance (*P* value) was *P* < 0.005 between dermcidin levels, systolic and diastolic pressures in hypertensive subjects before and after aspirin ingestion as determined by the Mann Whitney *U* test with the medians significantly different.

Hypertensive subjects (*n* = 74) were asked to swallow one 150 mg of aspirin with water after having a meal as described in Section 2. Both the blood pressures and dermcidin levels were determined before the ingestion of aspirin and 3 h after the ingestion of the compound.

## References

[1] Fuster V, Badimon J, Chesebro JH, Fallon JT (1996). Plaque rupture, thrombosis, and therapeutic implications. *Haemostasis*.

[2] Furman MI, Benoit SE, Barnard MR (1998). Increased platelet reactivity and circulating monocyte-platelet aggregates in patients with stable coronary artery disease. *Journal of the American College of Cardiology*.

[3] Ruberg FL, Loscalzo J (2002). Prothrombotic determinants of coronary atherothrombosis. *Vascular Medicine*.

[4] Libby P, Kasper DL, Braunwald E, Fauci AS, Hauser S, Longo D, Jameson JL (2005). Prevention and treatment of atherosclerosis. *Harrison’s Principles of Internal Medicine*.

[5] Maritim AC, Sanders RA, Watkins JB (2003). Diabetes, oxidative stress, and antioxidants: a review. *Journal of Biochemical and Molecular Toxicology*.

[6] Briones AM, Touyz RM (2010). Oxidative stress and hypertension: current concepts. *Current Hypertension Reports*.

[7] Sowers JR, Epstein M, Frohlich ED (2001). Diabetes, hypertension, and cardiovascular disease an update. *Hypertension*.

[8] Hopkins PN, Hunt SC, Wu LL, Williams GH, Williams RR (1996). Hypertension, dyslipidemia, and insulin resistance: links in a chain or spokes on a wheel?. *Current Opinion in Lipidology*.

[9] Sinha AK, Bhattacharya S, Acharya K, Mazumder S (1999). Stimulation of nitric oxide synthesis and protective role of insulin in acute thrombosis in vivo. *Life Sciences*.

[10] Sobel BE, Schneider DJ (2004). Platelet function, coagulopathy, and impaired fibrinolysis in diabetes. *Cardiology Clinics*.

[11] Chakraborty K, Sinha AK (2004). The role of insulin as an antithrombotic humoral factor. *BioEssays*.

[12] Ghosh R, Karmohapatra SK, Bhattacharyya M, Bhattacharya R, Bhattacharya G, Sinha AK (2011). The appearance of dermcidin isoform 2, a novel platelet aggregating agent in the circulation in acute myocardial infarction that inhibits insulin synthesis and the restoration by acetyl salicylic acid of its effects. *Journal of Thrombosis and Thrombolysis*.

[13] Ignarro LJ, Harbison RG, Wood KS, Kadowitz PJ (1986). Activation of purified soluble guanylate cyclase by endothelium-derived relaxing factor from intrapulmonary artery and vein: stimulation by acetylcholine, bradykinin and arachidonic acid. *Journal of Pharmacology and Experimental Therapeutics*.

[14] Ghosh R, Karmohapatra SK, Bhattacharya G, Kumar Sinha A (2010). The glucose-induced synthesis of insulin in liver. *Endocrine*.

[15] Messerli FH, Williams B, Ritz E (2007). Essential hypertension. *Lancet*.

[16] Chakraborty K, Khan GA, Banerjee P, Ray U, Sinha AK (2003). Inhibition of human blood platelet aggregation and the stimulation of nitric oxide synthesis by aspirin. *Platelets*.

[17] Laemmli UK (1970). Cleavage of structural proteins during the assembly of the head of bacteriophage T4. *Nature*.

[18] Chevallet M, Luche S, Rabilloud T (2006). Silver staining of proteins in polyacrylamide gels. *Nature Protocols*.

[19] Atha DH, Ingham KC (1981). Mechanism of precipitation of proteins by polyethylene glycols. Analysis in terms of excluded volume. *Journal of Biological Chemistry*.

[20] Perloff D, Grim C, Flack J (1993). Human blood pressure: determination by sphygmomanometry. *Circulation*.

[21] Jia L, Bonaventura C, Bonaventura J, Stamler JS (1996). S-nitrosohaemoglobin: a dynamic activity of blood involved in vascular control. *Nature*.

[22] Cox RD, Frank CW (1982). Determination of nitrate and nitrite in blood and urine by chemiluminescence. *Journal of Analytical Toxicology*.

[23] Engvall E, Perlmann P (1972). Enzyme-linked immunosorbent assay, Elisa. 3. Quantitation of specific antibodies by enzyme-labeled anti-immunoglobulin in antigen-coated tubes. *Journal of Immunology*.

[24] Matsudaira P (1987). Sequence from picomole quantities of proteins electroblotted onto polyvinylidene difluoride membranes. *Journal of Biological Chemistry*.

[25] Chakraborty S, Khan GA, Karmohapatra SK, Bhattacharya R, Bhattacharya G, Sinha AK (2009). Purification and mechanism of action of “cortexin”, a novel antihypertensive protein hormone from kidney and its role in essential hypertension in men. *Journal of the American Society of Hypertension*.

[26] Zimmerman R, Paluch U, Sprinzl M, Neupert W (1979). Cell-free synthesis of the mitochondrial ADP/ATP carrier protein of Neurospora crassa. *European Journal of Biochemistry*.

[27] Giaid A, Saleh D (1995). Reduced expression of endothelial nitric oxide synthase in the lungs of patients with pulmonary hypertension. *New England Journal of Medicine*.

[28] Smith JB, Willis AL (1971). Aspirin selectively inhibits prostaglandin production in human platelets. *Nature: New biology*.

[29] Karmohapatra SK, Chakraborty K, Kahn NN, Sinha AK (2007). The role of nitric oxide in aspirin induced thrombolysis in vitro and the purification of aspirin activated nitric oxide synthase from human blood platelets. *American Journal of Hematology*.

[30] Ferrannini E, Haffner SM, Stern MP (1990). Essential hypertension: an insulin-resistant state. *Journal of Cardiovascular Pharmacology*.

